# Two new species of Erythroneurini from China (Hemiptera, Cicadellidae, Typhlocybinae)

**DOI:** 10.3897/BDJ.9.e70141

**Published:** 2021-08-25

**Authors:** Xiao Yang, Guimei Luo, Yuehua Song

**Affiliations:** 1 School of Karst Science, Guizhou Normal University / State Key Laboratory Cultivation Base for Guizhou Karst Mountain Ecology Environment of China, Guiyang, Guizhou, 550001, China School of Karst Science, Guizhou Normal University / State Key Laboratory Cultivation Base for Guizhou Karst Mountain Ecology Environment of China Guiyang, Guizhou, 550001 China

**Keywords:** Cicadomorpha, Auchenorrhyncha, leafhopper, taxonomy, Karst

## Abstract

**Background:**

The leafhopper genus *Empoascanara* Distant, 1918 encompasses 81 species, most of which are distributed in Afrotropical, Oriental and Australian Realm. The leafhopper genu*s Kapsa* Dworakowska, 1972 encompasses 23 species, mainly known from the Oriental and Australian Realms.

**New information:**

Two new species of the leafhopper tribe Erythroneurini from Guizhou Province, China, *Empoascanaradichotomus* sp. nov. and *Kapsasinuose* sp. nov. are described and illustrated. Identification keys to the males of the genera *Empoascanara* and *Kapsa* in China are proposed.

## Introduction

The leafhopper genus *Empoascanara* Distant, 1918 was established with *Empoascanaraprima* Distant, 1918 as its type species. So far, 81 species of the genus *Empoascanara* have been reported, most of which are distributed in Africa, the Orient and Australia. The leafhopper genus *Kapsa* Dworakowska, 1972 was established with *Typhlocybafurcifrons* Jacobi, 1941 as its type species (Dworakowska 1972). So far, 24 species have been included in the genus, with nearly half recorded from India (Song and Li 2012). This genus also occurs in China, Sri Lanka, Vietnam and other Oriental countries. The genus *Kapsa* Dworakowska, 1972 includes the nominotypical subgenus and subgenus Rigida Cao & Zhang (Yang et al. 2013). In the present paper, two new species from Guizhou Province, China are described and illustrated below.

## Materials and methods

The specimens were obtained by sweep net and morphological terminology used in this work follows Dietrich (2005) and Song and Li (2013). Observation and drawings were made using an Olympus SZX16 and an Olympus BX53 microscopes. Habitus photos were taken using a KEYENCE VHX-5000 digital microscope. Body length is measured from the apex of vertex to the tip of the forewing. All specimens examined are deposited in the collection of the School of Karst Science, Guizhou Normal University, China (GZNU).

## Taxon treatments

### Empoascanara dichotomus sp. n.

B1624BA0-C327-5B9B-8A21-6C567E8A14B0

8D8871A7-7412-4CA1-B59B-C753326FC78F

#### Materials

- **Type status:**Holotype. **Occurrence:** recordedBy: Zhouwei Yuan and Chao Tan; individualCount: 1; sex: male; lifeStage: adult; **Taxon:** scientificName: *Empoascanaradichotomus*; order: Hemiptera; family: Cicadellidae; genus: Empoascanara; specificEpithet: *dichotomus*; **Location:** country: China; stateProvince: Guizhou; locality: Huajiang, Xiagu Village; **Event:** eventDate: 2019-05-2T15:25-0800; **Record Level:** collectionCode: insects; basisOfRecord: PreservedSpecimen
- **Type status:**Paratype. **Occurrence:** recordedBy: Zhouwei Yuan and Chao Tan; individualCount: 11; sex: female; lifeStage: adult; **Taxon:** scientificName: *Empoascanaradichotomus*; order: Hemiptera; family: Cicadellidae; genus: Empoascanara; specificEpithet: *dichotomus*; **Location:** country: China; stateProvince: Guizhou; locality: Huajiang, Xiagu Village; **Event:** eventDate: 2019-05-20T15:20-080; **Record Level:** collectionCode: Insect; basisOfRecord: PreservedSpecimen

#### Description

**Figure 1.**
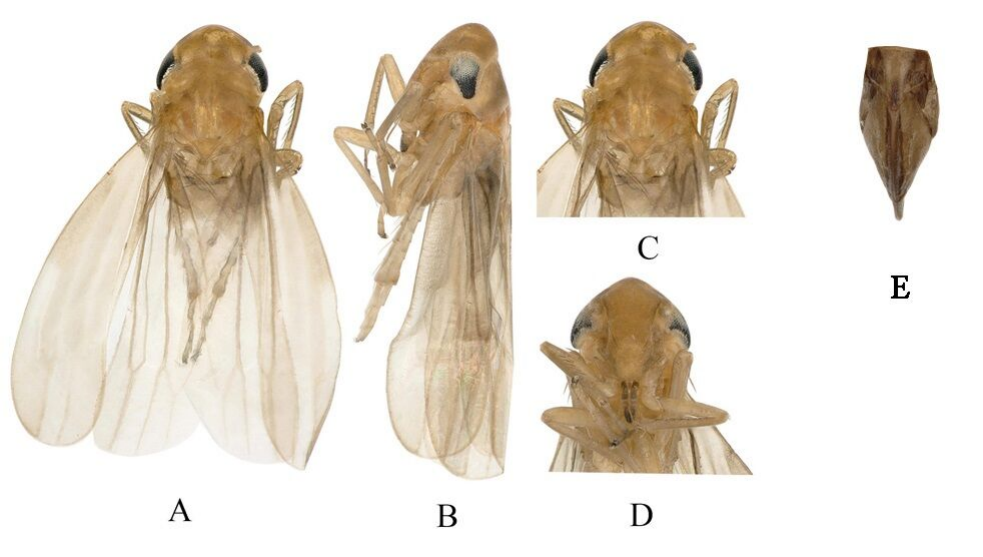
*Empoascanaradichotomus* sp. nov. **A.** Habitus, dorsal view; **B.** Habitus, lateral view; **C.** Head and thorax, dorsal view; **D.** Face; **E.** Female pygofer.

Body length male 2.1-2.3 mm; female 2.2-2.4 mm. Vertex brownish-yellow, with one irregular spot near anterior margin (Fig. 1A and C). Face brownish-yellow, relatively long, with pale yellow anteclypeus oval-shaped contrasting face colouration (Fig. 1D). Pronotum with large brownish-yellow markings running at middle (Fig. 1C). Scutellum brownish-yellow (Fig. 1C). Forewing and hindwing almost transparent (Fig. 1A). Abdominal apodemes small, not extended beyond hind margin of 3rd sternite (Fig. 3G).

#### Diagnosis

**Male genitalia.** Pygofer lobe with scattered microsetae at right edge. Dorsal pygofer appendage process without branch. Subgenital plate (Fig. 3B) broad sub-basally, provided with seven mid-length macrosetae on lateral surface centrally, several small fine setae scattered on apical area (Fig. 3B). Style apex with two terminal points, pre-apical lobe developed (Fig. 3D). Aedeagus with a pair of processes arising from apex of shaft; pre-atrium shorter than shaft, gonopore at subapex (Fig. 3E and F). Connective M-shaped (Fig. 3C).

#### Etymology

The specific name is derived from the Latin word “dichotomus” (bifurcated), referring to the bifurcated processes on the aedeagal shaft in the ventral view (Fig. 3E and F).

#### Taxon discussion

*Empoascanaradichotomus* is characterised with the morphology of the genus *Empoascanara* as follows:

Crown fore margin strongly produced and angulate medially or weakly produced, broadly rounded apically. Vertex usually with pair of dark pre-apical spots or with large median apical patch. Pronotum pale or almost entirely dark or with dark posterior margin. Forewing with venation of clavum obscure.

Male pygofer lobe rounded or angulate, without dorsal macrosetae, with sparse long fine setae. Pygofer dorsal appendage movably articulated, ventral appendage absent. Subgenital plates free, lateral margin with angulate sub-basal projection, with 2–4 basal macrosetae and distinct marginal sub-basal rigid setae forming continuous row or with marginal sub-basal rigid setae restricted to basolateral angle. Style pre-apical lobe prominent. Style apex smooth, slender or truncate and expanded, third point absent. Aedeagus dorsal apodeme not expanded in lateral view; shaft curved dorsad, smooth or denticulate distally; apex broadened, truncate or acuminate in ventral view; ventral processes placed basally, well separated from shaft. Connective median anterior lobe broad, arms short.

*Empoascanaradichotomus* is similar to *Empoascanarathomasi* Dworakowska, 1979; however, the new species has an aedeagus with a pair of processes on the apex instead of on the subapex and processes apex branched and the more chitinised aedeagus.

### Kapsa ramosis sp. n.

790F5DC7-4397-506C-9307-AA9338CC4EAC

4C975171-6573-409B-8971-F9E93CDB26B5

#### Materials

- **Type status:**Holotype. **Occurrence:** recordedBy: Zhouwei Yuan and Xiao Yang; sex: male; lifeStage: adult; **Taxon:** scientificName: *Kapsaramosis*; order: Hemiptera; family: Cicadellidae; genus: Kapsa; specificEpithet: *ramosis*; **Location:** country: China; stateProvince: Guizhou; county: Huajiang; **Event:** eventDate: 2019-06-20T11:11-0800; **Record Level:** collectionCode: Insects; basisOfRecord: PreservedSpecimen
- **Type status:**Paratype. **Occurrence:** recordedBy: Zhouwei Yuan and Xiao Yang; sex: 5 females; **Taxon:** scientificName: *Kapsaramosis*; order: Hemiptera; family: Cicadellidae; genus: Kapsa; specificEpithet: *ramosis*; **Location:** country: China; stateProvince: Guizhou; county: Huajiang; **Event:** eventDate: 2019-06-20T11:11-0800; **Record Level:** collectionCode: Insects; basisOfRecord: PreservedSpecimen

#### Description

**Figure 2.**
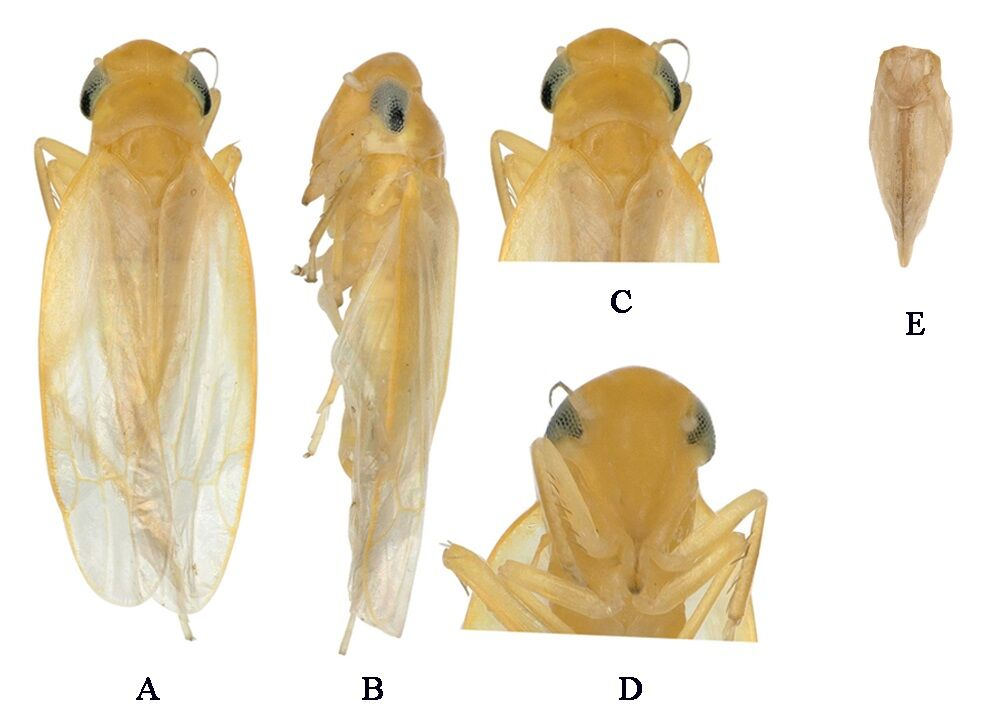
*Kapsaramosis* sp. nov. **A.** Habitus, dorsal view; **B.** Habitus, lateral view; **C.** Head and thorax, dorsal view; **D.** Face; **E.** Female pygofer.

**Figure 3.**
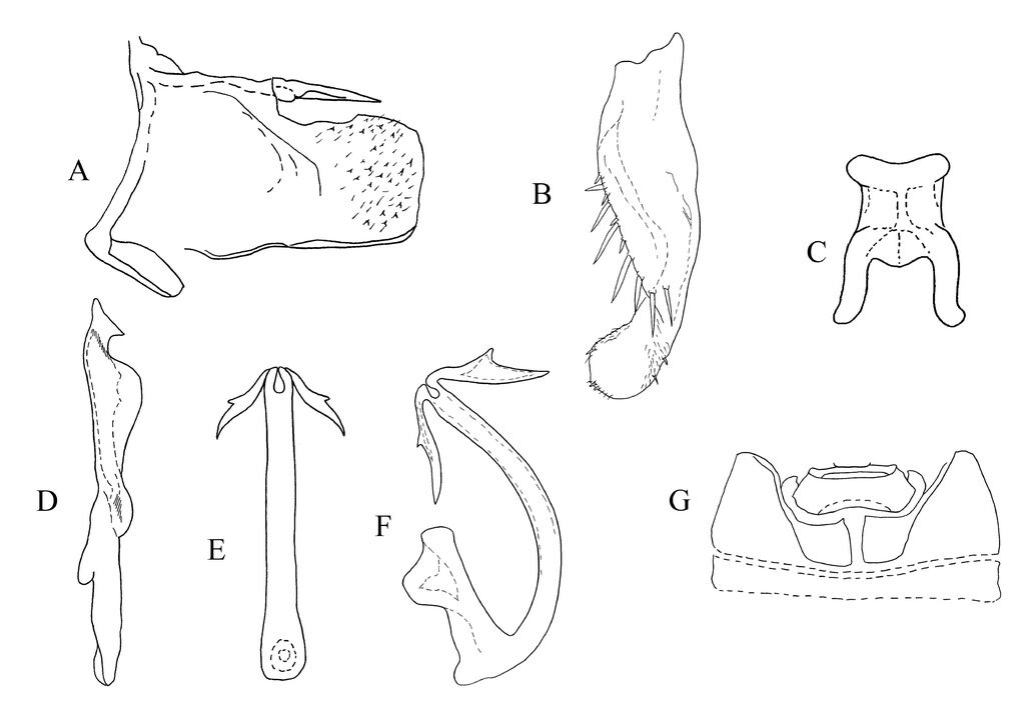
*Empoascanaradichotomus* sp. nov **A.** Pygofer, lateral view; **B.** Subgenital plate, dorsal view; **C.** Connective, ventral view; **D.** Style, lateral view; **E.** Aedeagus, ventral view; **F.** Aedeagus, lateral view; **G.** Abdominal apodemes, ventral view.

**Figure 4.**
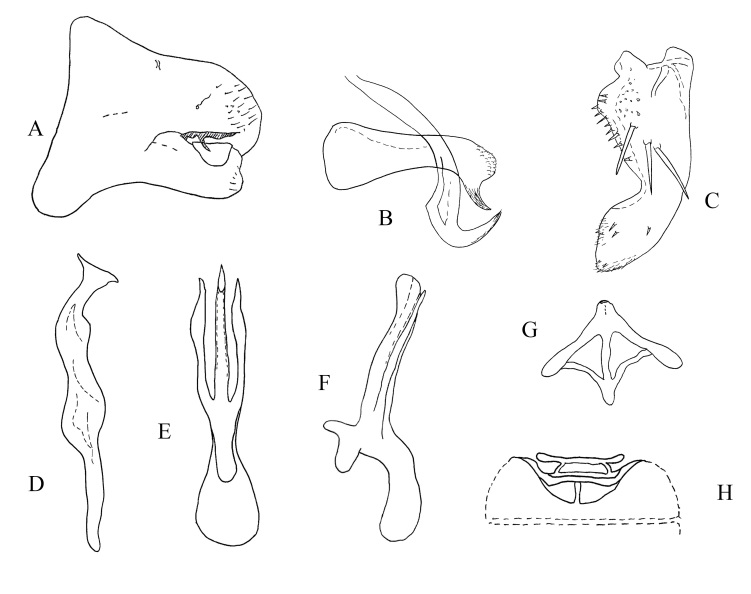
*Kapsaramosis* sp. nov. **A.** Pygofer, lateral view; **B.** Dorsal pygofer appendage, lateral view; **C.** Subgenital plate, lateral surface view; **D.** Style, lateral view; **E.** Aedeagus, ventral view; **F.** Aedeagus, lateral view; **G.** Connective, ventral view; **H.** Abdominal apodemes, ventral view.

Crown has anterior region yellow, while posterior area is yellow-milky, wider than pronotum. Face yellow, relatively long, with anteclypeus yellow (Fig. 2C). Pronotum milky yellow, without spots. Scutellum has anterior region yellow, while posterior area is yellow-milky. Border of forewings yellow, tinted with translucent centre (Fig. 2A and B). Male abdominal apodemes extended not beyond hind margin of 3rd sternite (Fig. 4H).

#### Diagnosis

**Male genitalia.** Pygofer lobe with scattered microsetae at right edge (Fig. 4C). Dorsal pygofer appendage process without branch. Subgenital plate (Fig. 4A) broad sub-basally, provided with three long macrosetae on lateral surface centrally, several small fine setae scattered on apical area (Fig. 4C). Style apex truncate, pre-apical lobe developed (Fig. 4D). Aedeagus long, with one pair of processes arising from base of shaft; pre-atrium shorter than shaft (Fig. 4E and F). Connective arms short (Fig. 4G).

#### Etymology

The specific name is derived from the Latin word “ramosis”, which means that species have two processes on the aedeagal shaft in the ventral side view (Fig. 4E).

#### Taxon discussion

The new species, *Kapsaramosis*, has the following morphological characters that places it within the subgenusKapsa (see also Song and Li 2012): Three macrosetae on the subgenital plate present (the subgenus Rigida has at least four macrosetae on the subgenital plate). Head is narrower than pronotum. Crown fore margin weakly produced, broadly rounded apically. Face depressed in profile, less than 45° from horizontal. Male anteclypeus narrow, depressed, as in female. Colour pattern brown. Vertex unicolorous or with pair of dark pre-apical spots or with median apical spot. Vertex mid-line pale or dark. Face without black spots anterodorsad of antennal pits. Anteclypeus pale, concolorous with rest of face or brown or black. Pronotum pale or with dark posterior margin. Mesonotum entirely pale or pale, with dark lateral triangles or entirely dark, apex concolorous with rest of mesonotum or apex dark, contrasting with adjacent pale areas. Thoracic ventre entirely pale or with dark mesosternum, remainder pale or entirely dark. Forewings without oblique vittae or with broken oblique vittae, without crossbands or with darkened apices, without numerous irregular red dots.

Male pygofer not extended to apex of subgenital plate. Pygofer lobe rounded. Pygofer ventro-apical membranous area well developed. Subgenital plate lateral margin with angulate sub-basal projection. Subgenital plates free. Style pre-apical lobe prominent. Style apex truncate and expanded or with 3 points. Aedeagus with pre-atrium shorter than shaft or with pre-atrium about as long as shaft. Aedeagus without dorsal process or with processes on dorsal apodeme. Connective median anterior lobe broad. Connective stem absent or very short, depressed.

*Kapsaramosis* is similar to *K.furcifrons* (Jacobi, 1941), but differs from it by having aedeagus with processes and the three macrosetae instead of five macrosetae on lateral surface centrally.

## Identification Keys

### Key to the males of *Empoascanara* in China (modified after Song and Li 2013)

**Table:** 

1	Aedeagal shaft without processes	2
–	Aedeagal shaft with processes	5
2	Aedeagus dorsal apodeme large, expanded	3
–	Aedeagus dorsal apodeme small, indistinct (Fig. 5A)	*E.alami* Dworakowska
3	Gonopore apical (Fig. 5B)	*E.sonani* Dworakowska
–	Gonopore subapical or median	4
4	Aedeagal shaft with apex broad; gonopore subapical (Fig. 5C)	*E.lata* Dworakowska & Pawer
–	Aedeagal shaft with apex narrow; gonopore median (Fig. 5D)	*E.kotoshonis* Dworakowska
5	Aedeagus with basal atrial processes	6
–	Aedeagus without basal atrial processes	12
6	Aedeagal shaft with apex bifurcate	7
–	Aedeagal shaft with apex not bifurcate	8
7	Basal atrial processes of aedeagus bifurcate (Fig. 5E)	*E.penta* Dworakowska
–	Basal atrial processes of aedeagus not bifurcate (Fig. 5F)	*E.limbata* Dworakowska
8	Aedeagal shaft short and pre-atrium long	9
–	Aedeagal shaft long and pre-atrium short	11
9	Basal atrial processes of aedeagus with lateral margin serrate apically (Fig. 5G)	*E.circumscripta* Chiang & Knigh
–	Basal atrial processes of aedeagus with lateral margin smooth apically	10
10	Pygofer dorsal appendage expanded at base, then narrowed abruptly and bifurcate at apex (Fig. 5H)	*E.nigrobimaculata* Dworakowska
–	Pygofer dorsal appendage broad and short, nearly equal to width and bifurcate at apex (Fig. 5I)	*E.sipra* Dworakowska
11	Gonopore apical on ventral surface (Fig. 5J)	*E.conchata* Song & Li
–	Gonopore sub-basal on ventral surface (Fig. 5K)	*E.mai* Dworakowska
12	Aedeagal shaft with paired apical processes	13
–	Aedeagal shaft with unpaired apical processes	14
13	Gonopore at apex (Fig. 5L)	*E.dwalata* Dworakowska
–	Gonopore at sub-apex	17
14	Pygofer dorsal appendage not branched apically (Fig. 5M)	*E.longiaedeaga* Song & Li
–	Pygofer dorsal appendage branched apically	15
15	Apical processes of aedeagal shaft lamella-like (Fig. 5Q)	*E.fumigata* Dworakowska
–	Apical processes of aedeagal shaft band-like	16
16	Aedeagal shaft with 3 asymmetrical apical processes (Fig. 5N)	*E.hongkongica* Dworakowska
–	Aedeagal shaft with 2 asymmetrical apical processes (Fig. 5O)	*E.maculifrons* Dworakowska
17	Aedeagus with a pair of not bifurcate processes arising from apex of shaft (Fig. 5P)	*E.mana* Dworakowska
–	Aedeagus with a pair of bifurcate processes arising from apex of shaft	*E.dichotomus* sp. nov.

### Key to the males of Kapsa (Kapsa) in China (modified after Song and Li 2012)

**Table:** 

1	Aedeagus with processes	2
–	Aedeagus without processes	5
2	Aedeagus with both basal and apical processes (Fig. 6A)	*K.quadrispina* Song & Li
–	Aedeagus either with basal processes or apical processes	3
3	Pygofer with dorsal appendage bifurcate	4
–	Pygofer with dorsal appendage not bifurcate (Fig. 6B)	*K.biprocessa* Song & Li
4	G onopore long (Fig. 6C)	*K.acuminata* Song & Li
–	G onopore moderately long	*K.ramosis* sp. nov.
5	Aedeagus with dorsal apodeme short and small, not expanded in lateral view (Fig. 6D)	*K.fangxianga* Song & Li
–	Aedeagus with dorsal apodeme large, greatly expanded in lateral view	6
6	Aedeagal shaft moderately long (Fig. 6E)	*K.arca* Song et al.
–	Aedeagal shaft moderately short	7
7	Aedeagal shaft slender and sinuate (Fig. 6G)	*K.dolka* Dworakowska
–	Aedeagal shaft broad and straight (Fig. 6F)	*K.suaoensis* Chiang & Knight

## Supplementary Material

XML Treatment for Empoascanara dichotomus

XML Treatment for Kapsa ramosis

## Figures and Tables

**Figure 5.**
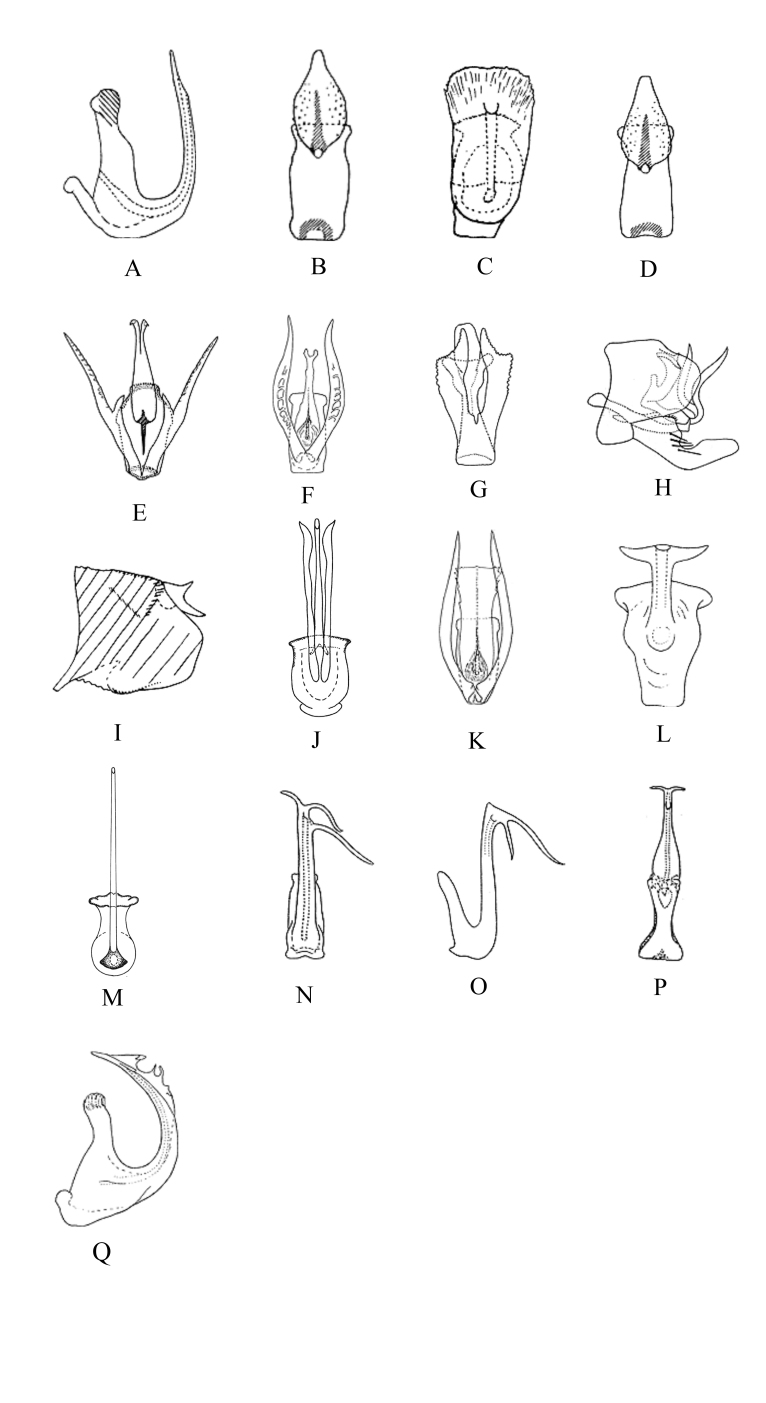
Мale genitalia of *Empoascanara* spp. **A** *E.alami*, Aedeagus, lateral view; **B** *E.sonani*, aedeagus, ventral view; **C** *E.lata*, aedeagus, ventral view; **D** *E.kotoshonis*, aedeagus, ventral view; **E** *E.penta*, аedeagus, ventral view; **F** *E.limbata*, aedeagus, ventral view; **G** *E.circumscripta*, aedeagus, ventral view; **H** *E.nigrobimaculata*, pygofer lobe, lateral view; **I** *E.sipra*, pygofer lobe, lateral view; **J** *E.conchata*, aedeagus, ventral view; **K** *E.mai*, aedeagus, ventral view; **L** *E.dewalata*, aedeagus, ventral view; **M** *E.longiaedeaga*, aedeagus, ventral view; **N** *E.hongkongica*, aedeagus, ventral view; **O** *E.maculifrons*, aedeagus, lateral view; **P** *E.mana*, aedeagus, ventral view; **Q** *E.fumigata*, aedeagus, lateral view.

**Figure 6.**
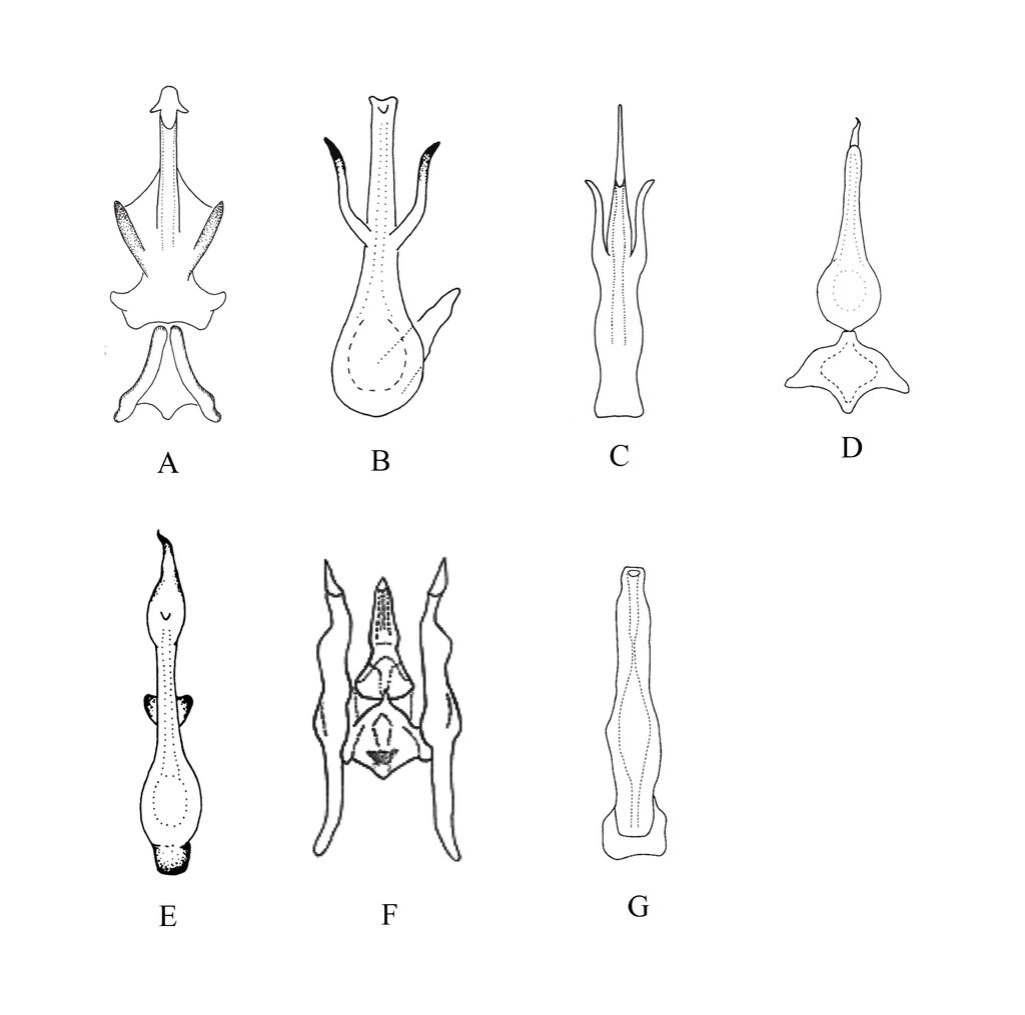
Мale genitalia of *Kapsa* spp. **A** *K.quadirispina*, aedeagus and connective, ventral view; **B** *K.biprocessa*, aedeagus, ventral view; **C** *K.acuminata*, аedeagus, ventral view; **D** *K.fangxianga*, aedeagus and connective, ventral view; **E** *K.arca*, аedeagus, ventral view; **F** *K.suaoensis*, aedeagus, connective and style, ventral view; **G** *K.dolka*, aedeagus, ventral view.
